# Efficient substructure feature encoding based on graph neural network blocks for drug-target interaction prediction

**DOI:** 10.3389/fphar.2025.1553743

**Published:** 2025-03-05

**Authors:** Guojian Deng, Changsheng Shi, Ruiquan Ge, Riqian Hu, Changmiao Wang, Feiwei Qin, Cheng Pan, Haixia Mao, Qing Yang

**Affiliations:** ^1^ School of Computer Science, Hangzhou Dianzi University, Hangzhou, China; ^2^ Department of Interventional Vascular Surgery, The Third Affiliated Hospital of Wenzhou Medical University, Wenzhou, China; ^3^ Hangzhou Institute of Advanced Technology, Hangzhou, China; ^4^ Jacob School of Engineering, University of California, San Diego, CA, United States; ^5^ Medical Big Data Laboratory, Shenzhen Research Institute of Big Data, Shenzhen, China; ^6^ School of General Education, Sanda University, Shanghai, China; ^7^ School of Automotive and Transportation Engineering, Shenzhen Polytechnic University, Shenzhen, China; ^8^ Department of Gastroenterology, Ruian People’s Hospital, The Third Affiliated Hospital of Wenzhou Medical University, Wenzhou, China

**Keywords:** graph neural network, drug discovery, graph representation learning, molecular substructure, drug-target interaction prediction

## Abstract

**Background:**

Predicting drug-target interaction (DTI) is a crucial phase in drug discovery. The core of DTI prediction lies in appropriate representations learning of drug and target. Previous studies have confirmed the effectiveness of graph neural networks (GNNs) in drug compound feature encoding. However, these GNN-based methods do not effectively balance the local substructural features with the overall structural properties of the drug molecular graph.

**Methods:**

In this study, we proposed a novel model named GNNBlockDTI to address the current challenges. We combined multiple layers of GNN as a GNNBlock unit to capture the hidden structural patterns from drug graph within local ranges. Based on the proposed GNNBlock, we introduced a feature enhancement strategy to re-encode the obtained structural features, and utilized gating units for redundant information filtering. To simulate the essence of DTI that only protein fragments in the binding pocket interact with drugs, we provided a local encoding strategy for target protein using variant convolutional networks.

**Results:**

Experimental results on three benchmark datasets demonstrated that GNNBlockDTI is highly competitive compared to the state-of-the-art models. Moreover, the case study of drug candidates ranking against different targets affirms the practical effectiveness of GNNBlockDTI. The source code for this study is available at https://github.com/Ptexys/GNNBlockDTI.

## 1 Introduction

Rapid identification of the interaction between drug compound and biological target is one of the crucial phases in drug discovery lim2021review. Traditional wet-lab drug-target interaction (DTI) screening is both expensive and time-consuming, with no assurance of success (Waring et al., 2015). Introducing computational methods to DTI identification for virtual screening can mitigate the issues associated with traditional approaches (Zhu, 2020). Specifically, machine learning, and more recently, deep learning, have significantly accelerated the validation cycle of new drug discovery while reducing costs and providing guidance for subsequent wet-lab experiments (Mak et al., 2024). In machine learning, the DTI prediction framework is defined as a dual-branch paradigm consisting of feature engineering for drugs and targets, respectively. Prediction models measure the possibility of interaction between a pair of drug targets by considering their feature representations (Zhang et al., 2017).

Drug compounds and target proteins were initially represented as a series of feature vectors and then calculated by a classifier to generate interaction scores (Cao et al., 2014). Compared to manual processing, automatic encoding of feature vectors using deep neural networks is a more efficient way (Hu et al., 2016; Wen et al., 2017). DeepDTA systematically demonstrated the superiority of drug/target embeddings learned by convolutional neural networks (CNNs) by comparison with the preprocessed feature matrices, even when extracted from the simple sequence inputs, drug SMILES strings, and protein amino acid sequences (Öztürk et al., 2018). Building on DeepDTA, GraphDTA employed molecular graphs instead of SMILES string for drug representation learning using graph neural networks (GNNs). The enhanced performance underscores the effectiveness of drug graph representations in the field of DTI predictions (Nguyen et al., 2021). Meanwhile, GraphDTA demonstrated that the 3-layer GNN used in their work could automatically recognize key substructures (functional groups) in drug graphs, even when no prior knowledge was given. Based on GNNs, DeepMGT-DTI extracted latent vectors from each of GNN layers and fused multi-scale latent vectors using the Transformer encoder for high-dimensional graph embeddings (Zhang et al., 2022). However, the shallow GNN-based framework is unable to adequately handle all of potential structural patterns that exist in molecular graphs, especially in complex molecules (Wieder et al., 2020). To capture high-dimensional topological features, DeepGLSTM input multi-hop adjacency matrices into GNNs, including the basic connectivity of chemical bonds and extended connections (Mukherjee et al., 2022). Despite its effectiveness, this method’s intricate graph embeddings pose challenges for biochemical interpretation. Alternatively, MGraphDTA proposed an ultra-deep GNN architecture with 27 layers to encode both local and global features of drug molecular graphs (Yang et al., 2022). The authors suggested that stacking sufficient GNN layers allows the receptive field of model to cover the entire graph, thus leveraging all available data in drug graphs. It is an insurance and effective way, but inevitably, the noise hidden in available information is difficult to distinguish. Contrary to the existence of methods that used GNNs to implicitly extract graph structural features, MSGNN-DTA chose to construct advanced molecular graphs for explicitly structural character encoding (Wang et al., 2023). MSGNN-DTA reconstructed the atom-level graphs into the motif graphs composed of subgraphs or substructures, incorporating complementary structural information. While subgraphs or substructures can be chemically explained as they determine molecule properties, predefined graph components face challenges due to generalization bias and the difficulty in accommodating all molecular variations.

To overcome the aforementioned challenges, we developed a novel model with enhanced encoding capabilities for molecular objects, named GNNBlockDTI. In processing drug data, we introduced the concept of the graph neural network block (GNNBlock), designed for the efficient extraction of local structural features. The GNNBlock comprises multiple GNN layers, which expand the model’s receptive field to capture substructural details across various scales. By stacking GNNBlocks, the substructural information within the entire graph can be collected and contribute to the overall structural properties. To ensure a more refined global representation and avoid the loss of important substructure features in our deep GNN-based architectures, we implemented a feature enhancement strategy and the gating units to improve the expressiveness of the learned structural features and filter out redundant information within each of GNNBlock, respectively.

In processing target proteins, we represented targets as both amino acid sequences and residue-level graphs to create a comprehensive protein embedding. In contrast to those methods with shallow fusion at the protein level (Yang et al., 2024; Sun et al., 2024), we embed sequential and spatial information simultaneously at the residue level for deep multimodal information fusion to achieve more expressive protein features. Considering only protein fragments around the binding pocket are involved in the real protein-ligand interaction process (Schenone et al., 2013), we focused on local fragment features in protein encodings. GNNBlockDTI utilizes CNNs and graph convolutional networks (GCNs) to encode sequences and graphs with the local convolutional operation, respectively. In summary, the main contributions of this paper are as follows:

• We proposed the concept of the GNNBlock as the fundamental component of our drug encoding module. The GNNBlock integrates multiple layers of GNN for a wide receptive field, which enables our model to concentrate on local structural characteristics within the molecular graph.
• We introduced a feature enhancement strategy within the GNNBlock to improve the expressiveness of node features in graphs. This strategy employs an “expansion-then-refinement” method. Initially, it maps features into a high-dimensional space and then refines them to retain relevant information.
• We incorporated gating units between GNNBlocks to manage the outputs of each block effectively. These gating units utilize a reset gate to filter out redundant information and an update gate to preserve essential features.
• We separately encoded the sequence and graph inputs of proteins from a localized perspective to achieve a multivariate protein representation that includes both sequence and spatial information. This local encoding strategy effectively reduces noise, allowing the model to concentrate on the specific residue fragments involved in drug-target binding.

## 2 Methods

**FIGURE 1:**
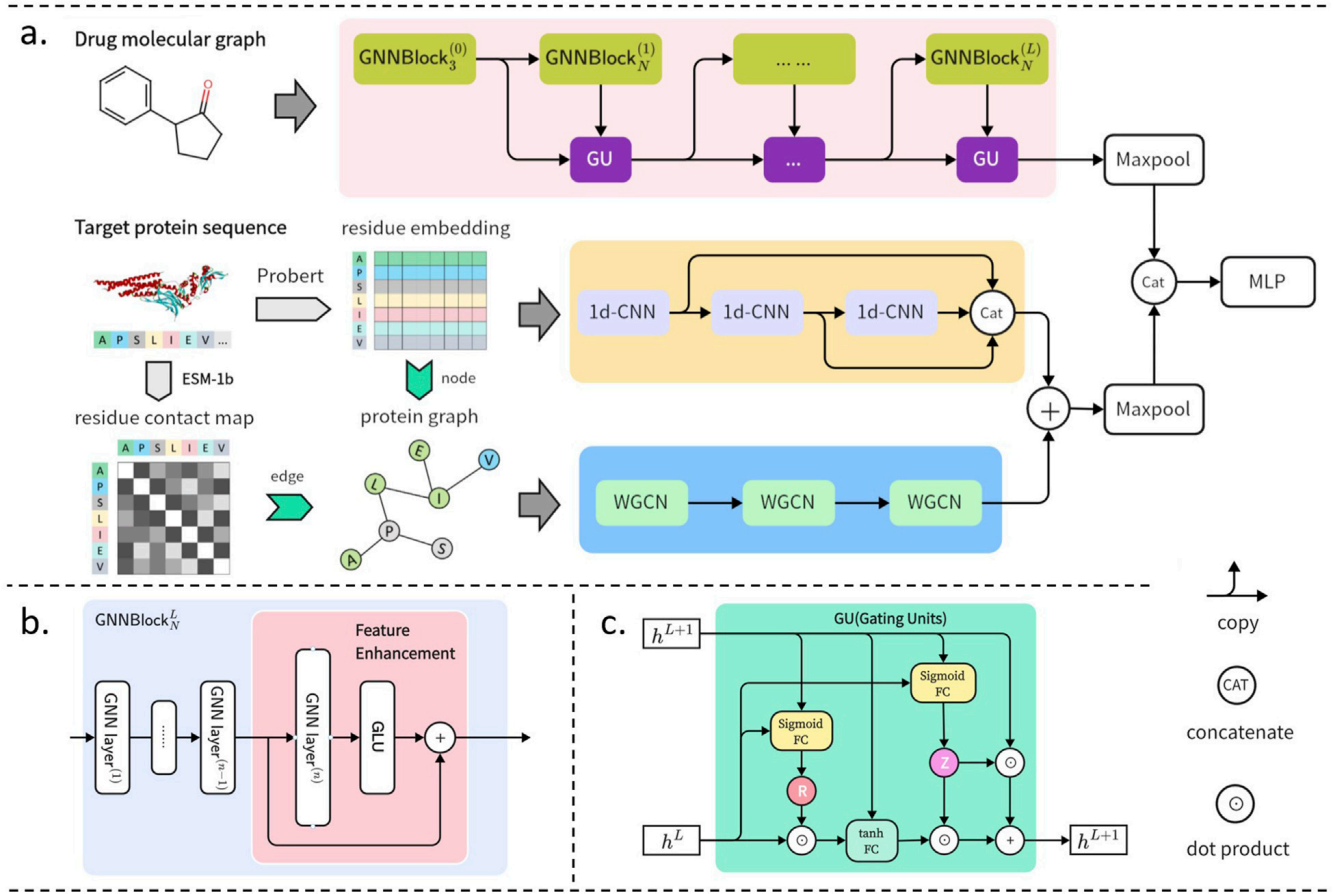
GNNBlockDTI for DTI prediction. **(A)** Overall framework of GNNBlockDTI. Drug graphs are encoded through the GNNBlock-based module. Targets sequences embedded by ProtBert are encoded through multi-scale CNNs, and target graphs constructed by ProtBert and ESM-1b are encoded through weighted GCNs. **(B)** GNNBlock incorporating the feature enhancement at the last GNN layer. **(C)** Details of gating units (GU) between GNNBlocks.

GNNBlockDTI takes drug graph representations and target dual representations as inputs and outputs the interaction probabilities of the drug-target pairs. The drug graph representations are encoded through the proposed GNNBlocks, and the target dual representations consisted of sequences and graphs are encoded through convolutional networks. Two obtained embeddings of drug-target are combined and input into a Multilayer Perceptron (MLP) classifier for DTI prediction. An overview of the proposed GNNBlockDTI is depicted in Figure 1A.

### 2.1 Drug molecular graph encoder

In this work, drugs were represented as molecular graphs that align well with their inherent characteristics and then encoded through the GNN-based module. Compared to SMILES string, graph representation of drug compound provides richer structural information for feature extraction. A drug graph is denoted by G=(V,E) , where the set of vertexes V corresponds to atoms, and the set of edges E is constructed from the connectivity of chemical bonds. Classical GNNs employ a message-passing mechanism for node feature encoding, which involves aggregating information from neighboring nodes and updating the representation of the node itself. We denote the i -th node vector at time step t as $x_{i}^{\left(t\right)}$ , updating $x_{i}^{\left(t\right)}$ to $x_{i}^{\left(t+1\right)}$ in the message passing phase through (Equation 1):

$$x_{i}^{\left(t+1\right)}=\sigma \left(F_{1}\left(x_{i}^{\left(t\right)}\right)+F_{2}\left(\underset{j\in N_{\left(i\right)}}{\sum }x_{j}^{\left(t\right)}\right)\right),$$ (1)

where σ(⋅) denotes activation function, $F_{1}\left(\cdot \right)$ and $F_{2}\left(\cdot \right)$ denote update function. $N_{\left(i\right)}$ is the set of neighbors of node i . The node vector $x_{i}^{\left(t+1\right)}$ at time step ( t +1) is obtained by combining its current vector $x_{i}^{\left(t\right)}$ with neighboring node vectors $x_{j}^{\left(t\right)}$ at time step t . After node feature learning by GNNs, the overall embedding of graph G is then read out from the node embedding matrix using a function with permutation invariance.

In this work, the initial node embeddings were constructed from various atomic properties, including Atomic Symbol, Formal Charge, Degree, IsAromatic, and IsInRing, the total dimension of a node embedding is 64. We utilized RDKit (Landrum, 2013) to extract these atomic properties and convert drug SMILES strings into molecular graphs.

#### 2.1.1 Substructural feature extraction based on GNNBlock

In drug feature encoding, we emphasized the local substructural properties of drug molecules, as these are crucial in determining the chemical characteristics. To capture substructural properties, we developed the GNNBlock based on GNN, which served as the foundational unit within our drug processing module. In addition to the GNNBlock, we incorporated a feature enhancement strategy to further encode the obtained structural features.

##### 2.1.1.1 GNNBlock

The message-passing mechanism allows each GNN layer to aggregate information from neighboring nodes within one hop. A single-layer GNN is inadequate for capturing the detailed local structure within a molecule. To address this limitation, we introduced the concept of GNNBlock, which combined multiple GNN layers into a single unit, thereby enhancing the receptive field for substructure encoding. A GNNBlock enables information aggregation from neighboring nodes within *N*-hop, where *N* depends on the number of GNN layers contained in GNNBlock. The definition of *N*-layer ${GNNBlock}_{N}$ is presented in (Equation 2):

$${GNNBlock}_{N}\left(x\right)={GNN}^{\left(n\right)}\left(\cdot \cdot \cdot {GNN}^{\left(1\right)}\left(x\right)\right),$$ (2)

where ${GNN}^{n}$ represents the *n*-th GNN layer in ${GNNBlock}_{N}$ . To ensure the feasibility of GNNBlock, the input and output channels of the intermediate GNN layers are kept consistent. By stacking several GNNBlocks, we could effectively capture most of the substructure properties hidden in the molecular graph.

##### 2.1.1.2 Feature enhancement

Based on the proposed GNNBlock, we implemented a feature enhancement strategy to re-encode the output from the GNNBlock into a high-dimensional embedding, effectively preserving learned crucial information. The structure incorporating feature enhancement within the GNNBlock is illustrated in Figure 1B. Feature enhancement follows an expansion-then-refinement approach. At the last GNN layer in GNNBlock, we increased the dimension of the node vector by doubling the output channels and extracted latent node vectors as in Equation 3:

$$h_{n-1}^{\left(L+1\right)},h_{n}^{\left(L+1\right)}={GNNBlock}_{N}^{\left(L+1\right)}\left(h^{\left(L\right)}\right),$$ (3)

where $h_{n}^{\left(L+1\right)}$ is the expanded latent vector at last GNN layer and $h_{n-1}^{\left(L+1\right)}$ is the latent vector at penultimate GNN layer in (*L*+1)-th ${GNNBlock}_{N}$ . $h^{\left(L\right)}$ is the input node vector from the previous GNNBlock. We applied a GLU activation function to refine this expanded vector and then conducted a residual connection between the two obtained latent vectors as in Equation 4:

$$h^{\left(L+1\right)}=\left(h_{n-1}^{\left(L+1\right)}+GLU\left(h_{n}^{\left(L+1\right)}\right)\right)\cdot d,$$ (4)

where *d* is a scaling factor. After feature enhancement, the resultant high-dimensional node vector $h^{\left(L+1\right)}$ is obtained.

In the specific implementation of GNNBlocks, we initially applied a fixed 3-layer ${GNNBlock}_{3}$ to extract the initial node embedding and then added multiple ${GNNBlock}_{N}$ to deeply mine the structural features, where N is a hyper-parameter. The deep GNNBlocks architecture enables substructural feature extraction across the entire graph to contribute to the overall properties of the drug molecular graph.

#### 2.1.2 Feature refinement based on gating units

We preferred to stack sufficient GNNBlocks to achieve an effective graph representation containing comprehensive structural information, but the deeper architecture also brought us new challenges. Excessive message-passing operations due to the deep architecture can easily lead to the accumulation of redundant information and noise within node embeddings, thereby affecting the performance of graph representation learning. Inspired by the framework of gated recurrent units (GRUs) Cho et al. (2014), we incorporated gating units between GNNBlocks to enable the model to concentrate effectively on essential features. The specifics of the gating units are illustrated in Figure 1C. We set up the reset gate **R** and update gate **Z** to filter the output of each GNNBlock, the gating units are calculated as follows in detail:

$$R=Sigmoid\left(h^{\left(L+1\right)}W_{1}^{\left(r\right)}+h^{\left(L\right)}W_{2}^{\left(r\right)}+b^{\left(r\right)}\right),$$ (5)

$$Z=Sigmoid\left(h^{\left(L+1\right)}W_{1}^{\left(z\right)}+h^{\left(L\right)}W_{2}^{\left(z\right)}+b^{\left(z\right)}\right),$$ (6)

where $h^{\left(L\right)}$ represents the node vector at L-th GNNBlock. The reset gate **R** decides whether to retain features from the previous layer, while the update gate **Z** determines the proportion of current versus previous features to be updated. We performed an element-wise multiplication between the reset gate **R** and the feature vector from the previous layer. Then we fused the previous information with the current features to obtain the hidden state, as depicted in Equation 7:

$$h^{\left(L+1\right)}=tanh\left(h^{\left(L+1\right)}W_{1}^{\left(h\right)}+\left(R⊙h^{\left(L\right)}\right)W_{2}^{\left(h\right)}+b^{\left(h\right)}\right),$$ (7)

where ⊙ denotes dot product operation. $W_{1}$ , $W_{2}$ , and b in Equations 5–7 are learnable weights shared across all GNNBlocks. Sigmoid(⋅) and tanh(⋅) denote two different activation functions. The node feature vectors were updated by Equation 8 using the update gate **Z**:

$$h^{\left(L+1\right)}=\left.\left(1-Z\right)⊙h^{\left(L\right)}+Z⊙h^{\left(L+1\right)}\right).$$ (8)

After multiple encoding and updating by our GNNBlock-based module, the obtained node feature matrix was sent to a max-pooling layer with permutation invariance to read out the drug molecular graph representations.

### 2.2 Target encoder

Considering the complexity of protein objects, we represented targets as both amino acid sequences and residue-level graphs simultaneously. The sequence and graph representation were encoded separately to embed sequential and spatial information, and then the two embeddings were fused to obtain a multi-dimensional representation of the target. Meanwhile, considering that only certain residue fragments within the protein binding pocket participate in the interaction with a ligand (drug), we focused on capturing local fragment embeddings using convolutional networks during protein feature encoding.

#### 2.2.1 Sequence embedding based on Multiscale-CNN

We first represented target proteins as sequences consisting of residue symbols, where the class of symbols is 21, including 20 regular residues and 1 unknown residue. Considering the limitations of one-hot encoding for residue symbols, we utilized ProtBert (Elnaggar et al., 2021) to embed sufficient semantic information of residues. A residue is originally embedded as a 30-dimensional vector, resulting in a protein sequence representation with dimensions *L* × 30, where *L* is the sequence length.

We implemented a 3-layer 1D-CNN with varying convolutional window sizes to encode the protein sequence at multiple scales. The latent vectors produced by each CNN layer are then combined to form the final protein sequence embedding.

#### 2.2.2 Graph embedding based on Weighted-GCN

Given the significance of spatial characteristics in determining protein properties, we also represented targets as the residue-level graphs, where the residues denote nodes and the 3D spatial distance between residues are used as edges. Considering the lack of structural data for some proteins, we utilized ESM-1b (Rives et al., 2021), which has been validated in DTI prediction works (Wang et al., 2023; Jiang et al., 2022), to acquire the contact map of residues for graph construction. A contact map M contains the spatial contact probability of any two residues in the protein. And a higher contact probability $M_{ij}$ indicates a higher contact probability between *i*-th residue and *j*-th residue. We represented a target graph as a weighted graph, where the weights of edges are directly assigned by the contact probabilities, thus emphasizing the residue pairs with a higher contact probability. Besides, the initial node embeddings in the protein graph were similarly encoded by ProtBert.

For the weighted graph representations, we utilized a 3-layer weighted GCN (WGCN) to capture spatially localized information effectively. WGCN considers the edge weights in the message passing phase, emphasizing neighboring nodes with high weight and neglecting those with low weight. A WGCN layer is defined by Equation 9.

$$h_{i}^{\left(l+1\right)}=\sigma \left(h_{i}^{\left(l\right)}W_{1}^{\left(l\right)}+\underset{j\in N\left(i\right)}{\sum }\frac{M_{ij}}{c_{ij}}h_{j}^{\left(l\right)}W_{2}^{\left(l\right)}\right),$$ (9)

where $h_{i}^{\left(l\right)}$ represents the *i*-th node vector at *l*-th WGCN layer. $W_{1}^{\left(l\right)}$ and $W_{2}^{\left(l\right)}$ are learnable weights at *l*-th layer. σ(⋅) donates an activation function. N(i) is the set of neighbors of node *i*, and $c_{ij}$ is the product of the square root of node degrees. $M_{ij}$ is the contact probabilities of *i*-th residue and *j*-th residue, which serve as the scalar weight on the edge from node *i* to node *j*.

#### 2.2.3 Multi-dimensional embedding for targets

Finally, we conducted an element-wise summation operation to fuse the obtained sequence embedding $T_{\mathrm{seq}}$ and graph embedding $T_{\mathrm{graph}}$ as in Equation 10:

$$T_{\mathrm{fused}}=\left(L_{2}\left(T_{\mathrm{seq}}\cdot W_{s}\right)+L_{2}\left(T_{\mathrm{graph}}\cdot W_{g}\right)\right)\cdot W_{t},$$ (10)

where $L_{2}\left(\cdot \right)$ donate L2-normalization operation. $W_{s}$ , $W_{g}$ , and $W_{t}$ are learnable weights. The resulting fused protein embedding $T_{\mathrm{fused}}$ was sent to the max-pooling layer to obtain a multivariate target representation.

### 2.3 Drug-target interaction prediction

In this work, we employed a GNNBlock-based module to encode drug graph embeddings with rich sub-structure properties and utilized convolutional networks to encode target sequence and graph representation from the local perspective. The obtained representations of drug-target were concatenated and then input into the MLP classifier for DTI prediction. The MLP consists of three linear transformation layers, each linear transformation layer followed by a nonlinear activation layer ReLU and a regularization layer dropout. We framed the DTI prediction as a binary classification task, where the prediction result indicated the probability of interaction between a pair of drug-target. The loss function was set to the binary cross-entropy (BCE) function as in Equation 11:

$$BCE=-\left[ylog\left(\hat{y}\right)+\left(1-y\right)log\left(1-\left(\hat{y}\right)\right)\right],$$ (11)

where y∈[0,1] is the binary label, and $\hat{y}$ is the prediction score.

### 2.4 Feature diversity in GNN-based model

In GNN-based models, the diversity of node features determines the effectiveness of graph representation learning. Inspired by Liu et al. (2020), we defined a metric for feature diversity of graphs to validate the advanced architecture of the GNNBlock-based module. We first defined a diversity metric $d_{ij}$ between the vectors of node *i* and node *j* with their Euclidean distance. A larger distance indicates a higher diversity between two nodes. Based on the diversity metric, we proposed a node diversity metric ${DS}_{i}$ for node *i* in a graph G(V,E) by Equation 12:

$${DS}_{i}=\frac{1}{n-1}\underset{j\in V,i\neq j}{\sum }d_{ij}.$$ (12)

And then, we could obtain the diversity metric ${DS}_{G}$ for the whole graph G using Equation 13:

$${DS}_{G}=\frac{1}{n}\underset{i\in V}{\sum }{DS}_{i}.$$ (13)

### 2.5 Gradient-weighted structural attribute mapping

Inspired by (Selvaraju et al., 2017; Yang et al., 2022), we perform a visualization method based on gradient information to visualize the learning process of the GNNBlock-based module. Specifically, we extracted the intermediate feature map H of each GNNblock, and then computed the gradient of the predicted scores P and obtained the average gradient of the feature map on k -th channel through Equation 14.

$$\alpha_{k}^{\left(n\right)}=\frac{1}{\left|V\right|}\underset{v\in V}{\sum }\frac{\partial P}{\partial H_{v,k}^{\left(n\right)}}$$ (14)

where $\alpha_{k}^{\left(n\right)}$ represents the average gradient at n -th GNNBlock, V represents vertex set. By performing a weighted sum of the channel weights and the intermediate feature map, the final gradient information $W\in R^{v}$ that can measure the importance of nodes in the drug graph is obtained by Equation 15.

$$W^{\left(n\right)}=\underset{k}{\sum }\alpha_{k}^{n}H_{k}^{\left(n\right)}$$ (15)

## 3 Experiments

### 3.1 Datasets

We selected BIOSNAP as the primary dataset for our experiments. Huang et al. (2021) obtained the same number of negative samples by random sampling to construct a balanced dataset, which served as the version in our work. To systematically and comprehensively evaluate our method, we also added two complementary datasets: DrugBank, and BindingDB. DrugBank is a balanced dataset obtained by Zhao et al. (2022) sampling from the public database DrugBank. BindingDB is an unbalanced independent dataset obtained by Huang et al. (2021) sampling from the public database BindingDB. The statistics for three datasets are summarized in Table 1.

**TABLE 1:** Summary of the BIOSNAP, DrugBank, and BindingDB datasets.

Dataset	Drugs	Targets	Pos interactions	Neg interactions
BIOSNAP	4,510	2,182	13,836	13,647
DrugBank	6,647	4,294	17,511	17,511
BindingDB	7,165	1,415	9,166	23,433

### 3.2 Experimental setup

#### 3.2.1 Hyper-parameter setting

The batch size was set to 64, and the dropout rate was set to 0.2. Adam optimizer with a 0.0005 learning rate was used to update model parameters. We searched for a selection of hyper-parameters for the GNNBlock-based module, the search results were presented in Section 3.6.

#### 3.2.2 Metrics

We selected Area Under the Receiver Operating Characteristic curve (AUROC) and Area Under the Precision-Recall curve (AUPR) as primary metrics, accuracy (ACC), precision (PR), recall (RE), and F1 score as complementary metrics. During the model training process, we saved the best-performing model on the validation set and evaluated its true performance on the test set.

#### 3.2.3 Dataset settings

To simulate real DTI prediction application scenarios, we compared our model with baselines under two data split strategies, random split, and unseen split:

• Random split: Drugs/targets randomly appear in the training set, validation set, and test set.
• Unseen split: Drugs/targets that appear in the test set will not appear in the training set and validation set.
• Cluster-based split: Minimize the similarity of drugs/targets in the trainingvalidation and test sets based on the unseen split setting. Drug similarity was measured by the Tanimoto coefficient of ECFP4 fingerprints, whereas target similarity was determined by comparing amino acid sequences through dynamic programming algorithms. Average-linkage hierarchical clustering was employed to partition drugs/targets into distinct clusters, ensuring optimal inter-cluster distances.

### 3.3 Baselines

We selected four typical state-of-the-art models as baselines for comparison. The details of baselines are described as follows. We followed the same hyper-parameter setting described in their papers. To be fair, we applied the same dataset setup to re-train the baseline for comparison.

#### 3.3.1 MolTrans

Huang et al. (2022) MolTrans proposed an FCS algorithm for input representation of drugs and targets, and embedded the contextual information using transformer encoder. They constructed an interaction matrix based on the substructures of a drug-target pair, and then applied a 2D-CNN for the interaction prediction.

#### 3.3.2 MGraphDTA

Yang et al. (2022) MGraphDTA utilized a 27-layer GCN with dense connectivity to encode drugs from local and global perspectives. The protein embeddings were obtained by multi-scale CNNs.

#### 3.3.3 DrugBAN

Bai et al. (2023) DrugBAN utilized GCN and CNN to process drug molecular graphs and protein 1D sequences, and proposed a deep bilinear attention network framework with domain adaptation to explicitly learn pair-wise local interactions between drugs and targets.

#### 3.3.4 BINDTI

Peng et al. (2024) BINDTI proposed a mixed model called ACmix to encode protein features by incorporating convolution and self-attention, and utilized GCN to obtain drug graph features. A bi-directional intention network was proposed to fuse drug and protein features based on multi-head attention and intention.

### 3.4 Comparison experiments

#### 3.4.1 Performance evaluation under random split setting

To test the performance of our proposed model, we first conducted a comparison experiment with baselines on three benchmark datasets under the random split setting. During specific works, we applied a five-fold cross-validation on two balanced datasets, BIOSNAP and DrugBank. The datasets were first divided into a training set and a test set in a 4: 1 ratio, and then followed random sampling from the training set to get the validation set, the ratio of training set, validation set, and test set is 7:1:2. In addition to balanced datasets, we also employed an unbalanced dataset BindingDB to test how well our model performs in the face of extreme data distributions. To ensure stability in the training process, we balanced positive and negative samples in the training set by random sampling. The specific sample sizes for the training, validation, and testing sets are 12,668, 6,644, and 13,289, respectively. The ratio of positive to negative is 1: 1 in the training set and 1: 6 in the validation and testing sets.

The results of comparison experiments are presented in Table 2. GNNBlockDTI outperformed the state-of-the-art models across all three datasets. Compared to the highest AUROC and AUPR achieved by baselines, our model showed improvements of 0.020 and 0.021 on BIOSNAP, 0.026 and 0.025 on DrugBank, and 0.011 and 0.045 on BindingDB. The exceptional performance across complementary metrics further underscores the effectiveness of GNNBlockDTI. However, the poor performance of absolute AUPR, PR, and F1 score on the BindingDB dataset suggested that our model’s performance will inevitably be affected by extreme data distributions.

**TABLE 2:** Comparison experiments under random split setting on three benchmark datasets.

Method	AUROC(std) ↑	AUPR(std) ↑	PR (std) ↑	RE (std) ↑	F1 score (std) ↑
BIOSNAP					
MolTrans	0.885 (0.001)	0.893 (0.004)	0.762 (0.013)	**0.869(0.008)**	0.817 (0.003)
MGraphDTA	0.902 (0.003)	0.905 (0.003)	0.837 (0.030)	0.804 (0.038)	0.819 (0.007)
DrugBAN	0.905 (0.004)	0.909 (0.005)	0.826 (0.015)	0.842 (0.010)	0.838 (0.003)
BINDTI	0.899 (0.002)	0.899 (0.003)	0.814 (0.018)	0.841 (0.009)	0.834 (0.003)
**GNNBlockDTI**	**0.925 (0.001)**	**0.930 (0.001)**	**0.855 (0.010)**	0.853 (0.011)	**0.854 (0.002)**
DrugBank					
MolTrans	0.878 (0.004)	0.887 (0.002)	0.786 (0.014)	0.834 (0.002)	0.809 (0.007)
MGraphDTA	0.875 (0.004)	0.877 (0.006)	0.827 (0.014)	0.764 (0.025)	0.794 (0.007)
DrugBAN	0.883 (0.003)	0.888 (0.003)	0.796 (0.016)	0.818 (0.006)	0.812 (0.003)
BINDTI	0.880 (0.001)	0.880 (0.003)	0.785 (0.014)	0.824 (0.006)	0.813 (0.001)
**GNNBlockDTI**	**0.909 (0.003)**	**0.913 (0.004)**	**0.833 (0.007)**	**0.841 (0.016)**	**0.837 (0.005)**
BindingDB					
MolTrans	0.912 (0.001)	0.617 (0.002)	0.460 (0.011)	0.874 (0.012)	0.605 (0.005)
DrugBAN	0.907 (0.002)	0.613 (0.004)	0.462 (0.004)	0.599 (0.002)	0.599 (0.002)
BINDTI	0.824 (0.004)	0.444 (0.004)	0.355 (0.019)	0.712 (0.007)	0.474 (0.006)
**GNNBlockDTI**	**0.923 (0.002)**	**0.662 (0.004)**	**0.467 (0.023)**	**0.895 (0.027)**	**0.614 (0.004)**

Note: Best performance and second-best performance are highlighted in bold and underlined, respectively. The format “mean (std)” represents the mean and standard deviation.

#### 3.4.2 Performance evaluation under unseen split setting

We verified the superiority of GNNBlockDTI on all three datasets using a routine random split setting. To test the robustness of GNNBlockDTI given different prediction scenarios, we also evaluated our approach on BIOSNAP under unseen drug/target settings. In the specific unseen drugs/targets settings, 20% drugs/targets were first randomly selected, and then all drug-target pairs associated with these drugs/targets formed the test set, the remaining pairs were used as training set and validation set. The unseen split settings reduce the influence of data leakage during the training process, avoiding over-optimistic results. Moreover, the unseen split settings more accurately reflect real-world conditions in drug discovery compared to the random split setting.

As demonstrated in Table 3, our model maintained strong and consistent performance in both unseen drug and unseen protein scenarios. Compared to the optimal performance of AUROC and AUPR in baselines, our approach achieved an improvement of 0.0053, 0.0086 under the unseen drug setting, and 0.1042, 0.0814 under the unseen target setting. Besides, the stable performances on BIOSNAP across two different split settings demonstrated our model’s generalization ability, especially under the unseen target setting. However, our model did not perform as well in the unseen drug setting as in the other split settings. The relatively poor performance of recall (RE) compared to baselines indicated the weakness of our model in identifying positive drug-target interactions. Although previous experiments have validated the effectiveness of GNNBlockDTI, the results here remind us that there is still room for improvement in our model, like the embedding of bilateral information used in MolTrans and DrugBAN.

**TABLE 3:** Comparison experiments under unseen split setting on BIOSNAP dataset.

Method	AUROC(std) ↑	AUPR(std) ↑	PR (std) ↑	RE (std) ↑	F1 score (std) ↑
BIOSNAP–unseen drug					
MolTrans	0.830 (0.001)	0.858 (0.005)	0.733 (0.117)	**0.804 (0.289)**	0.767 (0.005)
MGraphDTA	0.858 (0.003)	0.882 (0.002)	0.826 (0.006)	0.711 (0.013)	0.764 (0.003)
DrugBAN	0.883 (0.006)	0.896 (0.006)	0.799 (0.008)	0.802 (0.017)	0.811 (0.007)
BINDTI	0.868 (0.003)	0.887 (0.004)	0.828 (0.008)	0.773 (0.012)	0.797 (0.003)
**GNNBlockDTI**	**0.888 (0.002)**	**0.904 (0.003)**	**0.844 (0.009)**	0.781 (0.023)	**0.812( 0.009)**
BIOSNAP–unseen target					
MolTrans	0.667 (0.046)	0.689 (0.050)	0.507 (0.071)	**0.917 (0.132)**	0.653 (0.008)
MGraphDTA	0.751 (0.004)	0.773 (0.006)	0.776 (0.015)	0.503 (0.028)	0.611 (0.013)
DrugBAN	0.670 (0.016)	0.667 (0.021)	0.690 (0.037)	0.371 (0.027)	0.483 (0.014)
BINDTI	0.649 (0.008)	0.665 (0.008)	0.699 (0.025)	0.394 (0.014)	0.504 (0.009)
**GNNBlockDTI**	**0.855 (0.006)**	**0.854 (0.002)**	**0.820 (0.010)**	0.672 (0.021)	**0.739 (0.008)**

Note: Best performance and second-best performance are highlighted in bold and underlined, respectively. The format “mean (std)” represents the mean and standard deviation.

**FIGURE 2:**
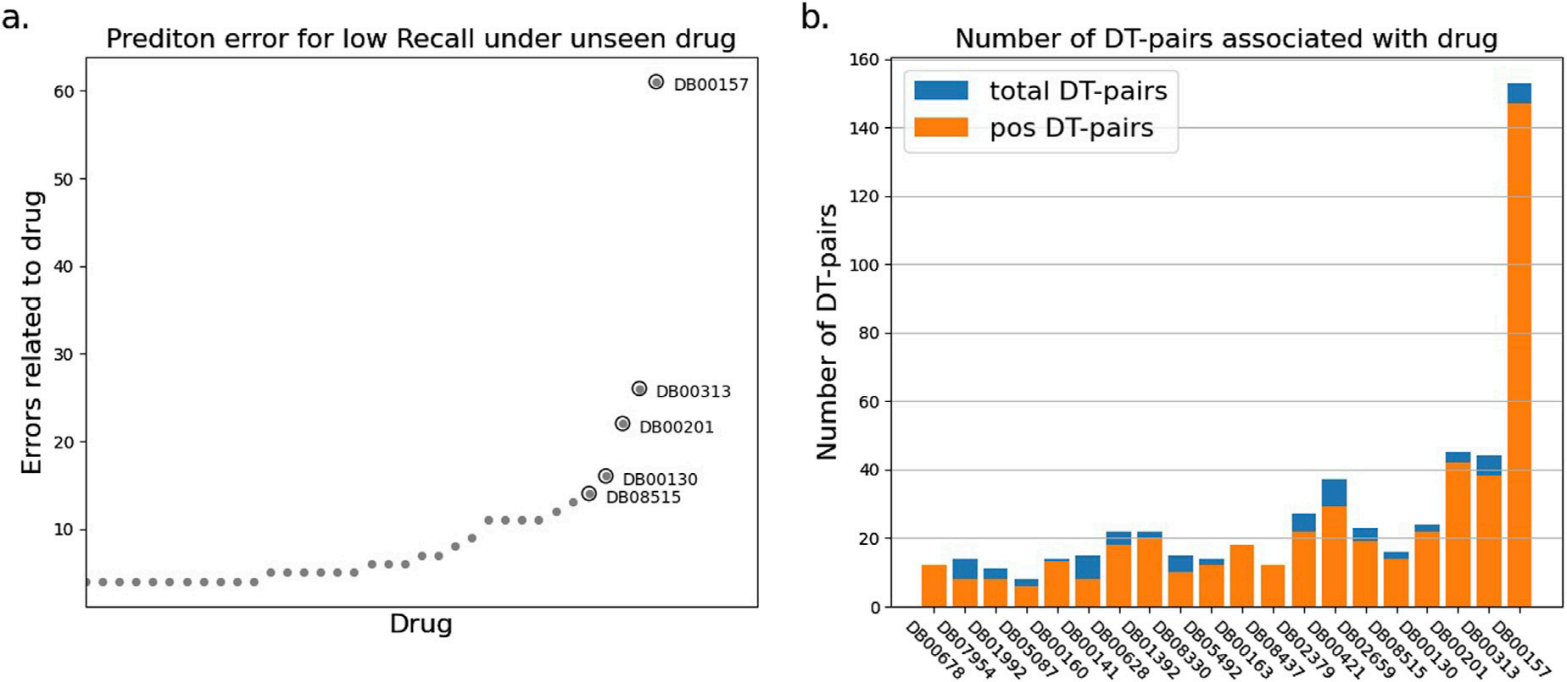
Statistics of drug-target pairs contributing to RE errors in the BIOSNAP test set under unseen drug setting. **(A)** Number of drug-target pairs (DT-pairs) per drug leading to RE errors. **(B)** Positive and negative proportions of DT pairs associated with “error” drug.

**FIGURE 3:**
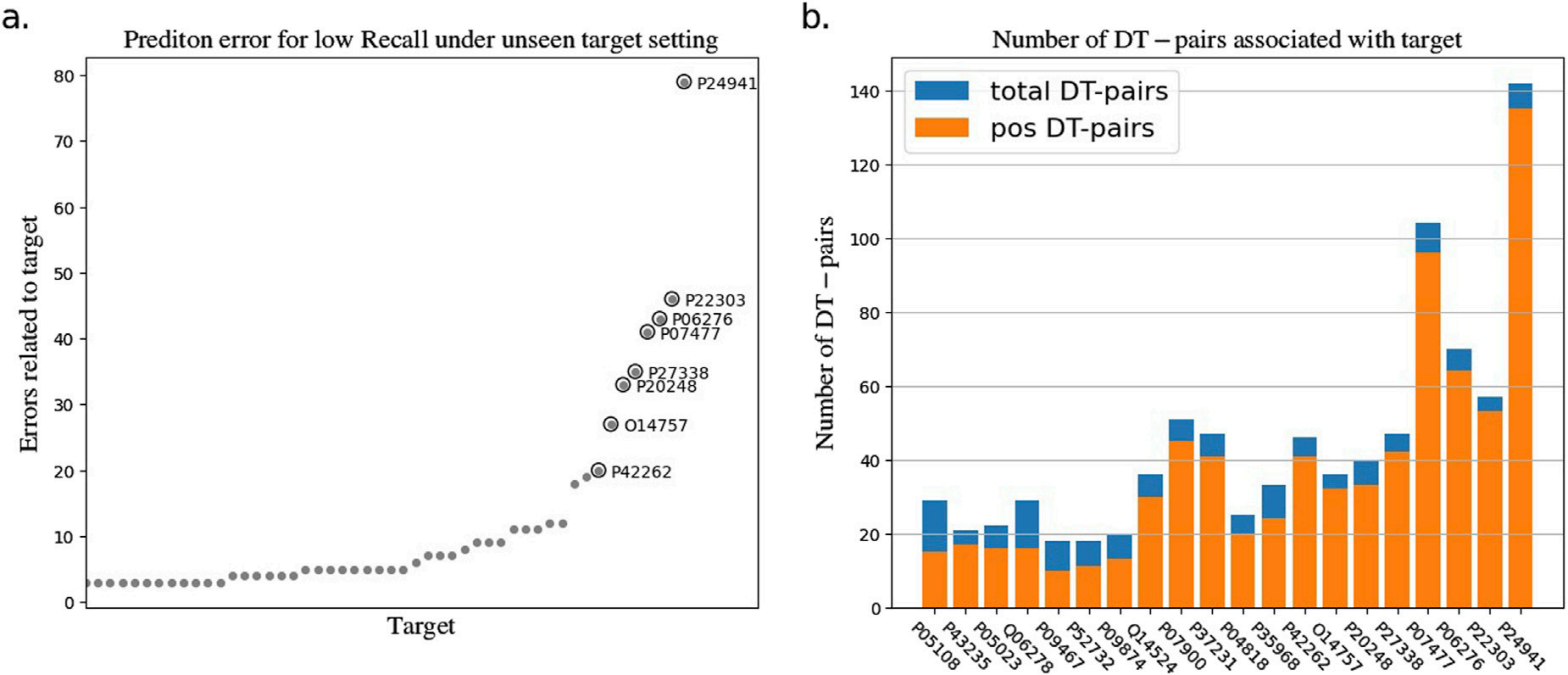
Statistics of drug-target pairs contributing to RE errors in the BIOSNAP test set under unseen target setting. **(A)** Number of drug-target pairs (DT-pairs) per target leading to RE errors. **(B)** Positive and negative proportions of DT pairs associated with “error” target.

To explore the reasons for the model’s poor performance on the RE metrics, we statistically analyzed the drug-target pairs that led to RE errors in the test set which those pairs with true label and false predicted label. The statistics show that a few drugs/targets contribute disproportionately to large recall errors, as shown in Figures 2, 3. After analyzing the data composition of the two test sets, we identified a one-sided data imbalance as causing the above phenomenon. Drugs/targets that contribute large recall errors tend to have more associated drug-target pairs, and there is an uneven positive and negative ratio for the drug-target pairs related to these error-related drugs/targets.

#### 3.4.3 Performance evaluation under cluster-based split setting

Acknowledging the limitations of the unseen split setting in simulating real-world prediction scenarios, we introduced an advanced split strategy rooted in hierarchical clustering that incorporates sample similarity across different sets while maintaining the principles of the unseen split setting. By minimizing structural/sequential similarities of drugs/targets between training and test sets, we further evaluated our model’s robustness and generalization capabilities. The comparative experimental results of clustering partitions on the BIOSNAP dataset are presented in Table 4. Our model consistently demonstrates superior performance, achieving optimal results across all evaluation metrics while maintaining minimal performance degradation compared to the other two split settings.

**TABLE 4:** Comparison experiments under cluster-based split setting on BIOSNAP dataset.

Method	AUROC(std) ↑	AUPR(std) ↑	PR (std) ↑	RE (std) ↑	F1 score (std) ↑
BIOSNAP–drug clustering					
MolTrans	0.790 (0.012)	0.777 (0.009)	0.772 (0.010)	0.613 (0.026)	0.683 (0.008)
MGraphDTA	0.756 (0.011)	0.762 (0.014)	0.724 (0.021)	0.623 (0.019)	0.670 (0.015)
DrugBAN	0.779 (0.005)	0.769 (0.007)	0.776 (0.022)	0.542 (0.017)	0.639 (0.028)
BINDTI	0.791 (0.009)	0.793 (0.004)	0.770 (0.013)	0.615 (0.011)	0.684 (0.023)
**GNNBlockDTI**	**0.809 (0.008)**	**0.812 (0.003)**	**0.801 (0.011)**	**0.628 (0.017)**	**0.699 (0.007)**
BIOSNAP–target clustering					
MolTrans	0.682 (0.012)	0.708 (0.017)	0.650 (0.020)	0.542 (0.013)	0.591 (0.010)
MGraphDTA	0.763 (0.005)	0.771 (0.012)	0.758 (0.009)	0.570 (0.031)	0.651 (0.015)
DrugBAN	0.618 (0.013)	0.622 (0.008)	0.685 (0.022)	0.421 (0.027)	0.434 (0.016)
BINDTI	0.603 (0.012)	0.6 (0.014)	0.653 (0.024)	0.471 (0.029)	0.483 (0.017)
**GNNBlockDTI**	**0.841 (0.009)**	**0.847 (0.007)**	**0.827 (0.012)**	**0.651 (0.013)**	**0.728 (0.006)**

Note: Best performance and second-best performance are highlighted in bold and underlined, respectively. The format “mean (std)” represents the mean and standard deviation.

### 3.5 Ablation study

The inclusion of GNNBlock is crucial to our model’s optimal performance. To illustrate the effectiveness and necessity of each module in the design of the GNNBlock, we conducted a series of ablation experiments on the BIOSNAP dataset. In addition, we also validated the superiority of spatial information embedding in target encoding.

The results of ablation experiments are detailed in Table 5. FE represents the feature enhancement mechanism in GNNBlocks, GU represents the gating units incorporated between GNNBlocks. When we removed both the feature enhancement mechanism and gating units, which is equivalent to the removal of GNNBlock as well. From Table 4, it is evident that the feature enhancement mechanism, gating units, GNNBlock, and target graph encoding play a significant role in contributing to the overall performance of GNNBlockDTI.

**TABLE 5:** Ablation experiments about Feature Enhancement (FE), Gating Units (GU), and target graph.

Method	AUROC(std) ↑	AUPR(std) ↑	ACC(std) ↑	F1score (std) ↑
w/o FE	0.906 (0.002)	0.910 (0.002)	0.837 (0.006)	0.842 (0.002)
w/o GU	0.907 (0.001)	0.907 (0.001)	0.837 (0.002)	0.835 (0.001)
w/o GU&FE	0.879 (0.003)	0.870 (0.002)	0.814 (0.002)	0.817 (0.003)
w/o target graph	0.908 (0.002)	0.911 (0.002)	0.835 (0.001)	0.836 (0.001)
GNNBlockDTI	0.925 (0.001)	0.930 (0.001)	0.853 (0.002)	0.854 (0.002 h)

Note: The format “mean (std)” represents the mean and standard deviation.

### 3.6 Hyper-parameter optimization for GNNBlock

As the core of our model, we focused on the specific architectural implementation of the GNNBlock, which is designed for efficient substructure encoding based on GNN. Shallow GNN architectures are capable of substructure recognition but are not complete. While deep GNN architectures enable a comprehensive collection of structural features across the entire graph, the interspersed noise will affect the encoding of key structural features. Meanwhile, the deep GNN architectures are constrained by the inherent limitations of over-smoothing or over-squashing of node features. To optimize our model’s performance, we explored various combinations of hyper-parameters about the GNN layers included in the GNNBlock-based module, including the number *N* of GNN layers in GNNBlock, and the number *L* of GNNBlocks.

**FIGURE 4:**
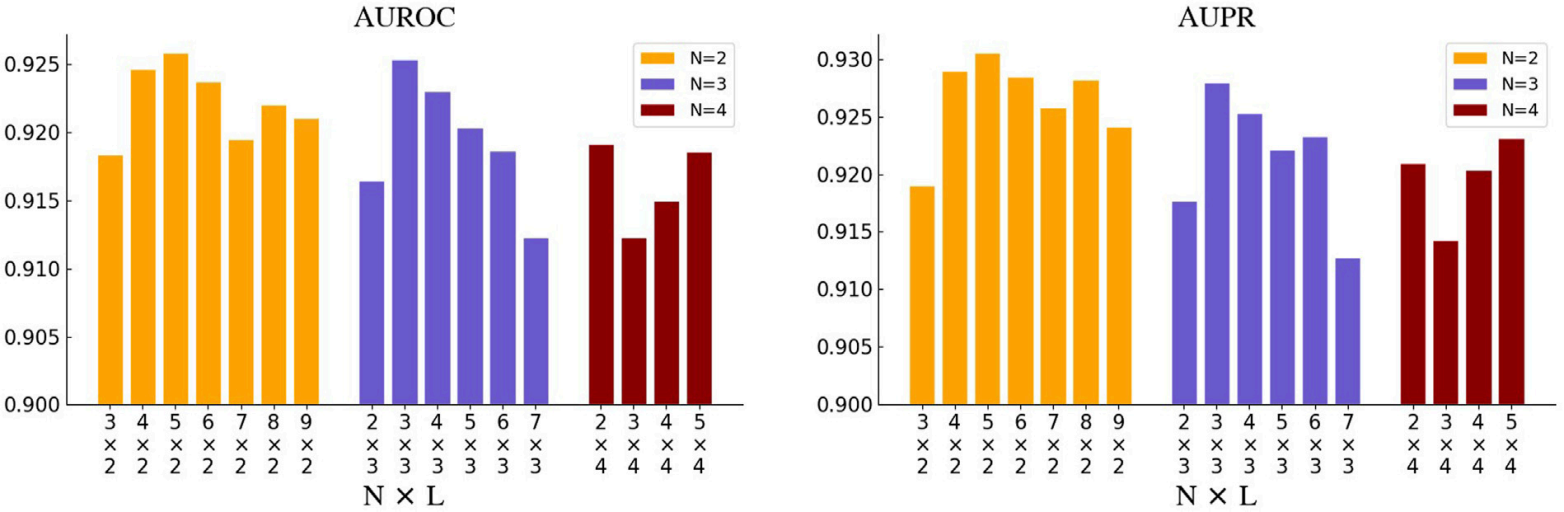
The performance of GNNBlockDTI with various combinations of the number *N* of GNN layers in GNNBlock and the number *L* of GNNBlocks.

**FIGURE 5:**
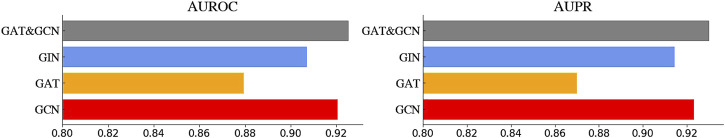
The performance of GNNBlockDTI with four GNN variants in GNNBlock, including GCN, GAT, GIN, GAT&GCN.

We first selected *N* from [2, 3, 4] and adjusted *L* accordingly to ensure the total number of GNN layers does not exceed 21. The search results of *N* and *L* are illustrated in Figure 4. When *N* is set to 2 or 3, the model achieves the optimum where the total number of the GNN layers reaches around 10. While the model performance continues to decline as *L* increases. And when *N* is set to 4, the model performs unexplainably worse. In addition, we also tried different GNN variants as the base unit of GNNBlock, including GCN, GAT, GIN, and GCN&GAT, where GAT&GCN indicates the use of GAT before the last layer and the GCN at the last layer in GNNBlock. The comparison results of four GNN variants are displayed in Figure 5.

Ultimately, our optimized GNNBlock-based module is structured with *L* = 5 GNNBlocks, each containing *N* = 2 GNN layers. The GNN type used in GNNBlock is the GAT&GCN.

### 3.7 Model interpretability

#### 3.7.1 Visualization of drug-target fused feature

**FIGURE 6:**
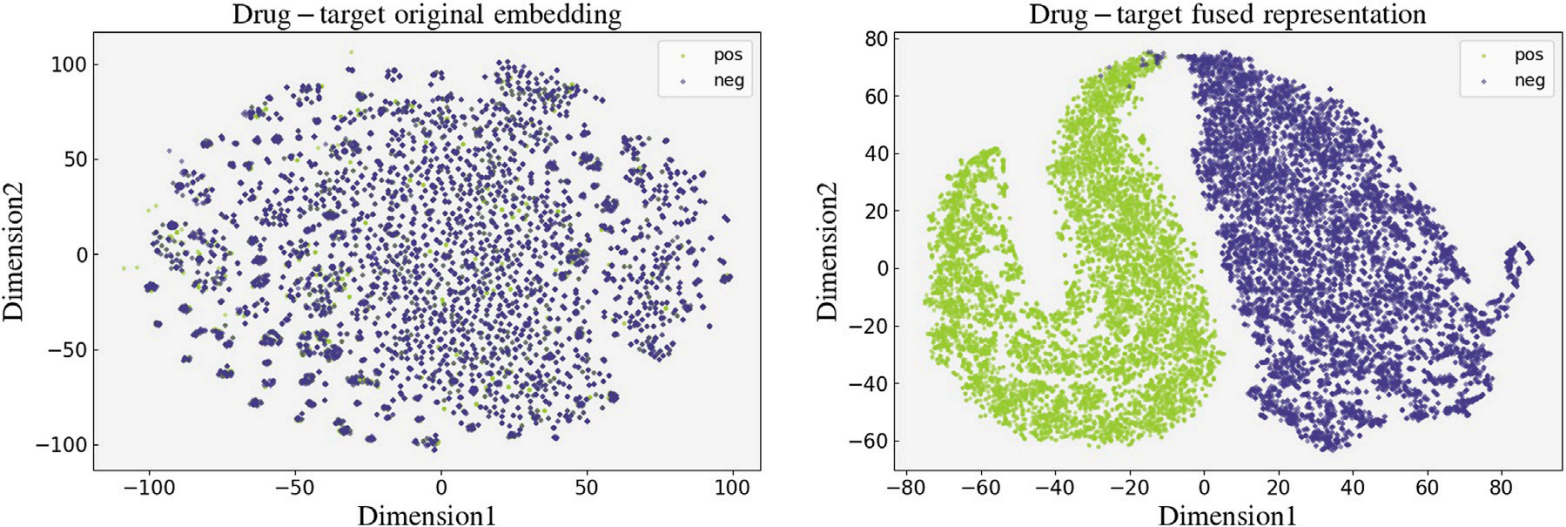
Visualization of the original embedding (left) and the fused feature (right) learned by GNNBlockDTI of drug-target pairs.

To verify the effectiveness of our model, we performed a visualization for the learned high-dimensional drug-target fused features. We first extracted the drug-target fused feature representations of the 19,112 drug-target pairs in the BIOSNAP training set and then downscaled them to 2D data using the t-SNE (van der Maaten and Hinton, 2008) algorithm. The visualization results of the 2D fused representations with labels are presented in Figure 6, we did the same visualization for the original embedding of drug-target pairs as a control. The distinct separation between true and false interactions in this visualization attests to the accuracy and validity of our model’s feature encoding.

#### 3.7.2 Feature diversity based on GNNBlock

**FIGURE 7:**
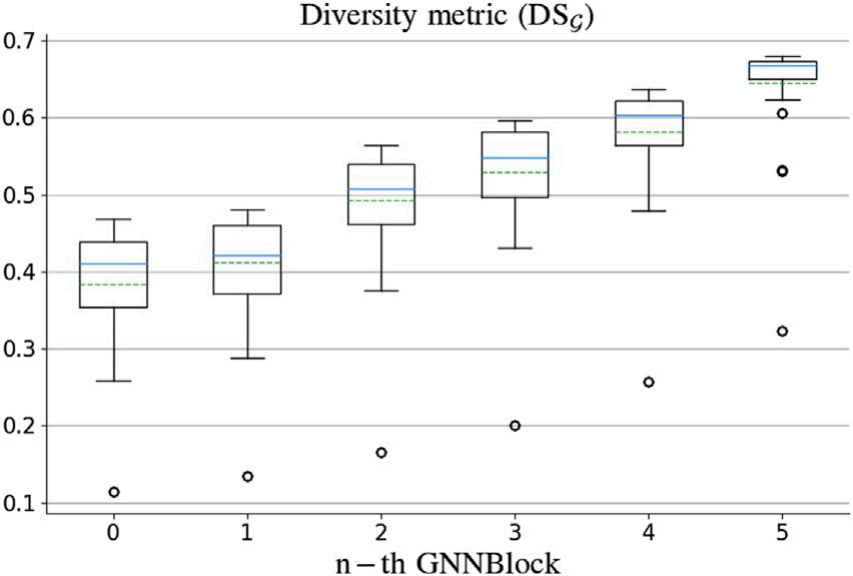
The diversity metric of graph features learned by each GNNBlock.

To explore the impressive performance of the GNNBlock-based architecture, we extracted the learned latent features from each GNNBlock for diversity assessment. We selected 47 complex drugs with a higher number of atoms from the BIOSNAP dataset for feature diversity assessment. Figure 7 presents the diversity metric values of graphs from each GNNBlock. The diversity of node features increases with deeper GNNBlocks, which indicates our GNNBlock-based architecture is capable of effective feature learning for molecular structure. Meanwhile, the results show that the GNNBlock is hardly affected by the inherent limitations of the message-passing mechanism in GNN, like feature over-smoothing or over-squashing.

#### 3.7.3 Local structural pattern recognized by GNNBlock

**FIGURE 8:**
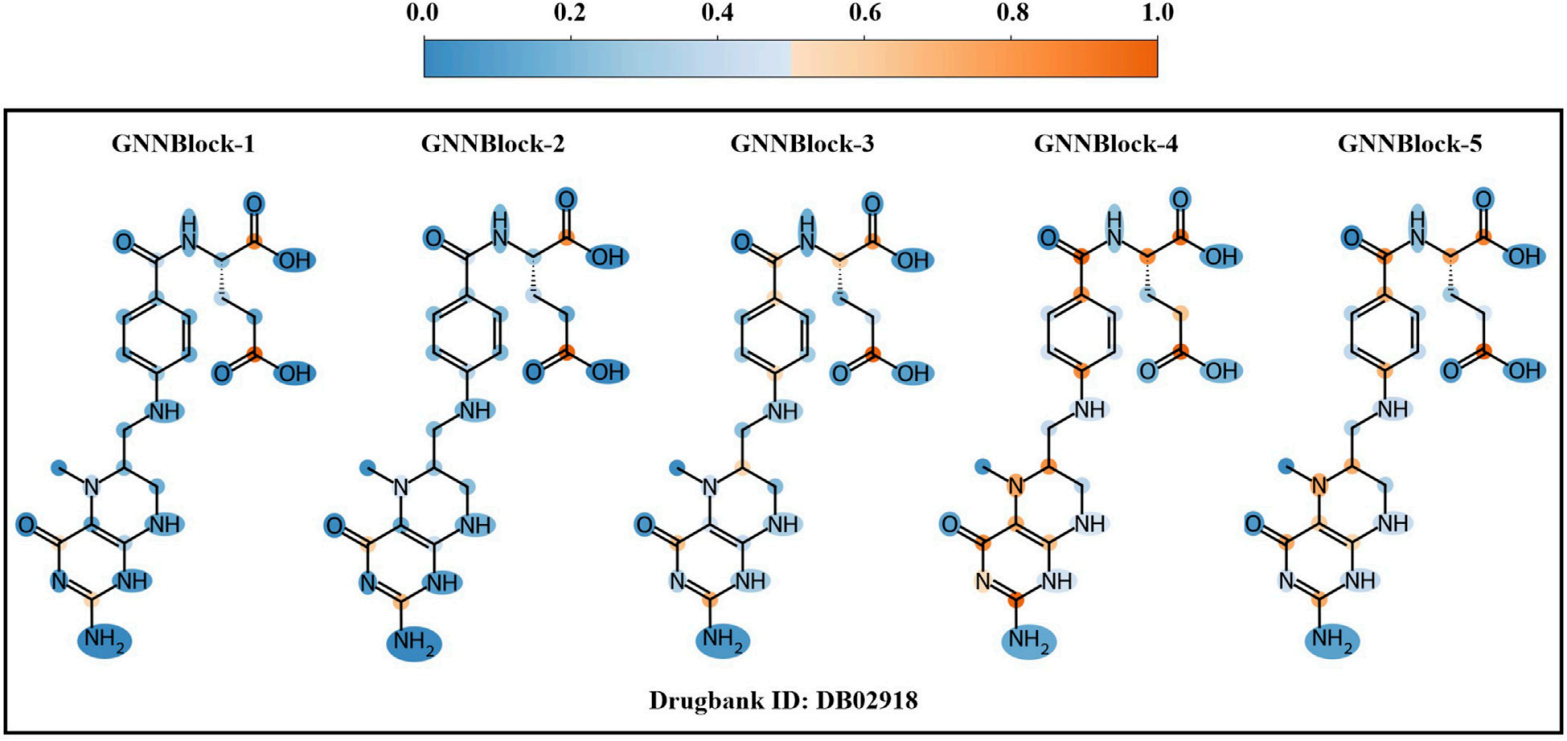
Visualization of node importance based on gradient information for each GNNBlock.

Despite the outstanding performance of our model, we do not know if the model will work as we expected during the learning and prediction process. The entire model remains a black box. To rationalize the GNNBlock-based module, we apply a visualization method based on gradient information to visualize the learning process of the GNNBlock-based module. We selected the optimal model structured with 5 GNNBlocks achieved in Section 3.6 as the visualization model. The encoding process of the GNNBlock-based module on the drug graph is shown in Figure 8. As GNNBlock goes deeper, our GNNBlock-based module is capable of selectively focusing on important local structural pattern in the molecule graph and capturing the overall structural properties of the entire graph.

### 3.8 Case study

To validate the effectiveness of our model in practical drug discovery scenarios, we selected two targets for candidate ranking prediction, including adenosine 5′-monophosphoramidase HINT1 (P49773), and Receptor for adenosine (P30542). For the task of predicting drug candidate rankings, we created candidate libraries with a ratios of 1:100 of true to false candidates for each of the two targets. True candidates interact with the target, whereas false candidates do not. The model we used in drug candidate rankings was training on BIOSNAP dataset, where all drug-target pairs involving a query drug/target in the case study were removed. The ranking results are detailed in Table 6, where we presented the top 10 candidates based on their predicted interaction probability with the target. In the case of P49773, all 5 true candidates appeared in the top 10. In P30542, 7 out of 11 true candidates were within the top 10, while the remaining 4 were ranked 18th, 30th, 32nd, and 46th.

**TABLE 6:** Top 10 drug candidates which were predicted to interact with P49773 and P30542.

Rank	P49773		P30542	
	DrugBank ID	True Label	DrugBank ID	True Label
1	DB01752	0	DB08844	0
2	DB00131	1	DB01223	1
3	DB02183	0	DB00277	1
4	DB01972	1	DB00201	1
5	DB02483	1	DB00806	1
6	DB04099	1	DB01303	1
7	DB03708	0	DB07733	0
8	DB03349	0	DB04638	0
9	DB00627	1	DB00651	1
10	DB02162	0	DB00824	1

In addition, to validate the effectiveness of GNNBlockDTI in the drug repositioning task, we also performed the target candidate ranking task for two selected drugs DB08875 and DB00647 with the same settings as drug candidate rankings. As presented in Table 7, In DB08875, all 5 true candidates appeared in the top 10. In DB00647, 6 out of 8 true candidates were within the top 10, while the remaining 2 were ranked 22nd and 31st. These results clearly demonstrate the reliability and effectiveness of our model in realistic drug discovery.

**TABLE 7:** Top 10 target candidates which were predicted to interact with DB00647 and DB08875.

Rank	DB00647		DB08875	
	Uniprot ID	True Label	Uniprot ID	True Label
1	P10632	1	P35968	1
2	P08684	1	Q15788	0
3	P01859	0	P08581	1
4	P10635	1	P08684	1
5	P24462	1	P03372	0
6	P20813	0	P11712	1
7	P11712	1	Q8IZS8	0
8	P46098	0	P07949	1
9	P35372	1	P00533	0
10	P06276	0	O00329	0

To further illustrate the advantages of our proposed model, we compared GNNBlockDTI with the traditional molecular docking method Autodock-vina (Trott and Olson, 2010) in the drug candidate ranking task. We selected 20 targets for drug candidate ranking with the same settings as above. AUROC, enrichment factor (EF), and ROC enrichment (Re) (Jain and Nicholls, 2008) were used as evaluation metrics to assess the model’s performance. The average metric across all targets is presented in Table 8. We performed the drug candidate ranking with autodock-vina based on the calculated binding energy, and the threshold for the positive and the negative was set at -5 kcal/mol. The results show that our model performs better in the case study compared to the molecular docking method.

**TABLE 8:** Comparison results of drug candidate ranking.

	AUROC	1%EF	5%EF	1%Re	5%Re
Autodock-vina	0.771	17.756	45.812	8.142	5.723
GNNBlockDTI	0.943	38.604	62.754	45.688	14.292

Note: The percentage before Re is the given threshold of FPR.

## 4 Discussion

In this DTI prediction work for drug discovery, we constructed a robust and comprehensive GNN-based drug molecular encoding framework and provided a rational approach for target feature encoding. Our drug encoding framework is manifested as a two-stage feature extraction strategy. GNNBlock is proposed for capturing local substructures critical to the exhibited properties of drug molecules. The feature enhancement mechanism and gating units are introduced into deep GNNBlocks to coordinate substructural features across the graph and achieve comprehensive structure characteristics. We also considered the biological nature of the drug-target binding process and proposed a localized encoding strategy focused on residue fragments in the target encoding. In addition, we embed semantic and spatial information at the residue level for a multidimensional vector of residues, to achieve an effective target representation. Comparative experiments confirmed the superiority of our overall model, and we also evaluated each of the modules separately in the subsequent analysis.

We conducted a series of searches to investigate the capability of GNNBlock used for substructural capture in Section 3.6. When the number of GNN layers included in a GNNBlock was set to 2 or 3, experimental results were broadly in line with our expectations. The concept of GNNBlock enhances GNN’s encoding capability. Before reaching the optimum, our model’s performance gradually improved as the depth of the GNNBlock increased. When the criticality was reached, the performance continued to decline, which is determined by the inherent limitations of the message-passing mechanism (Liu et al., 2020). However, when we adopted GNNBlock with 4 or higher GNN layers, the model’s performance behaved inexplicably worse. There is still much more to the combination of molecular graph data and graph neural networks that are waiting to be explored in DTI prediction. Besides, considering the unsatisfactory encoding capability of message-passing neural networks with deeper architectures, the current challenges also include novel frameworks for effective graph representation learning. The Graph Transformer based on self-attention mechanism processing topological information provides us with a direction (Wu et al., 2024; Rampášek et al., 2024), which is included in our future work.

We selected the molecular graphs as the unique drug encoding channel, without including the drug SMILES string, although previous work had confirmed the contribution of SMILES input to DTI prediction. Models cannot infer the underlying structural features from the associated symbols alone but perform a similarity correlation encoding (molecules with similar sequence compositions have more associations). These similarity-based features may even affect the performance of our graph encoding module. Actually, similar problems had already arisen in the early stages of our work. On the contrary, we adopted bimodal representation in the encoding engineering of the target, as the commonly used sequence representations contain limited valid information. So we embedded 3D structural information in the form of topological graphs to complement our encoding strategy.

The introduction of bilateral information can improve the stability of the model in the face of extreme data distributions, according to the experimental results presented in Section 3.4. Our model demonstrated outstanding performance under a routine random split setting due to the powerful feature encoding capability, while the gap between GNNBlockDTI and some of baselines under the unseen split and clustering-based settings became close. The representation embedded with bilateral correlation information coped well with such extreme data distributions, as implemented in the well-performing baselines, MolTrans (Huang et al., 2021) and DrugBAN (Bai et al., 2023). And this aspect will also included in our future work.

## 5 Conclusion

In this study, we proposed a novel model named GNNBlockDTI for drug-target interaction prediction. GNNBlockDTI thoroughly considered the natural properties of the drug and targets molecular objects, as well as the essence of the drug-target interaction. In drug encoding, we proposed the concept of GNNBlock based on GNN and introduced the feature enhancement mechanism as well as the gating units for substructural feature learning. In protein encoding, we focused on capturing localized features by utilizing 1D-CNN-based modules for sequential features and GCN-based modules for spatially localized features. We conducted a series of experiments to evaluate the effectiveness of GNNBlockDTI across three benchmark datasets. The results demonstrated that GNNBlockDTI outperformed the state-of-the-art models. Furthermore, realistic case studies highlighted our model is a powerful and robust tool in drug discovery.

## Data Availability

Publicly available datasets were analyzed in this study. This data can be found here: https://github.com/Ptexys/GNNBlockDTI.

## References

[B1] Bai P. Miljković F. John B. Lu H. (2023). Interpretable bilinear attention network with domain adaptation improves drug–target prediction. Nat. Mach. Intell. 5, 126–136. 10.1038/s42256-022-00605-1

[B2] Cao D.-S. Zhang L.-X. Tan G.-S. Xiang Z. Zeng W.-B. Xu Q.-S. (2014). Computational prediction of drug target interactions using chemical, biological, and network features. Mol. Inf. 33, 669–681. 10.1002/minf.201400009 27485302

[B3] Cho K. van Merrienboer B. Çaglar G. Bahdanau D. Bougares F. Schwenk H. (2014). Learning phrase representations using rnn encoder–decoder for statistical machine translation. Available at: https://arxiv.org/abs/1406.1078.

[B4] Elnaggar A. Heinzinger M. Dallago C. Rehawi G. Wang Y. Jones L. (2021). Prottrans: toward understanding the language of life through self-supervised learning. IEEE Trans. Pattern Analysis Mach. Intell. 44, 7112–7127. 10.1109/TPAMI.2021.3095381 34232869

[B5] Hu P.-W. Chan K. C. You Z.-H. (2016). “Large-scale prediction of drug-target interactions from deep representations,” in 2016 international joint conference on neural networks (IJCNN) (Vancouver: IEEE), 1236–1243. 10.1109/IJCNN.2016.7727339

[B6] Huang K. Xiao C. Glass L. M. Sun J. (2021). Moltrans: molecular interaction transformer for drug-target interaction prediction. Bioinformatics 37, 830–836. 10.1093/bioinformatics/btaa880 33070179 PMC8098026

[B7] Jain A. N. Nicholls A. (2008). Recommendations for evaluation of computational methods. J. computer-aided Mol. Des. 22, 133–139. 10.1007/s10822-008-9196-5 PMC2311385 18338228

[B8] Jiang M. Wang S. Zhang S. Zhou W. Zhang Y. Li Z. (2022). Sequence-based drug-target affinity prediction using weighted graph neural networks. BMC genomics 23, 449. 10.1186/s12864-022-08648-9 35715739 PMC9205061

[B9] Landrum G. (2013). Rdkit: a software suite for cheminformatics, computational chemistry, and predictive modeling. Greg Landrum 8, 5281. Available at: https://zenodo.org/badge/latestdoi/10009991.

[B10] Liu M. Gao H. Ji S. (2020). “Towards deeper graph neural networks,” in Proceedings of the 26th ACM SIGKDD international conference on knowledge discovery and data mining (New York: Association for Computing Machinery), 338–348. 10.1145/3394486.3403076

[B11] Mak K.-K. Wong Y.-H. Pichika M. R. (2024). Artificial intelligence in drug discovery and development. Drug Discov. Eval. Saf. Pharmacokinet. assays, 1461–1498doi. 10.1007/978-3-031-35529-5_92

[B12] Mukherjee S. Ghosh M. Basuchowdhuri P. (2022). “Deepglstm: deep graph convolutional network and lstm based approach for predicting drug-target binding affinity,” in Proceedings of the 2022 SIAM international conference on data mining (SDM) (Philadelphia: SIAM), 729–737. 10.1137/1.9781611977172.82

[B13] Nguyen T. Le H. Quinn T. P. Nguyen T. Le T. D. Venkatesh S. (2021). Graphdta: predicting drug–target binding affinity with graph neural networks. Bioinformatics 37, 1140–1147. 10.1093/bioinformatics/btaa921 33119053

[B14] Öztürk H. Özgür A. Ozkirimli E. (2018). Deepdta: deep drug–target binding affinity prediction. Bioinformatics 34, i821–i829. 10.1093/bioinformatics/bty593 30423097 PMC6129291

[B15] Peng L. Liu X. Yang L. Liu L. Bai Z. Chen M. (2024). Bindti: a bi-directional intention network for drug-target interaction identification based on attention mechanisms. IEEE J. Biomed. Health Inf. PP, 1–11. 10.1109/JBHI.2024.3375025 38457318

[B16] Rampášek L. Galkin M. Dwivedi V. P. Luu A. T. Wolf G. Beaini D. (2024). “Recipe for a general, powerful, scalable graph transformer,” in Proceedings of the 36th international conference on neural information processing systems, 15. Red Hook: Curran Associates Inc. 10.5555/3600270.3601324

[B17] Rives A. Meier J. Sercu T. Goyal S. Lin Z. Liu J. (2021). Biological structure and function emerge from scaling unsupervised learning to 250 million protein sequences, Proc. Natl. Acad. Sci. U. S. A. 118, e2016239118. 10.1073/pnas.2016239118 33876751 PMC8053943

[B18] Schenone M. Dančík V. Wagner B. K. Clemons P. A. (2013). Target identification and mechanism of action in chemical biology and drug discovery. Nat. Chem. Biol. 9, 232–240. 10.1038/nchembio.1199 23508189 PMC5543995

[B19] Selvaraju R. R. Cogswell M. Das A. Vedantam R. Parikh D. Batra D. (2017). “Grad-cam: visual explanations from deep networks via gradient-based localization,” in Proceedings of the IEEE international conference on computer vision, 618–626.

[B20] Sun Y. Li Y. Y. Leung C. K. Hu P. (2024). ingnn-dti: prediction of drug–target interaction with interpretable nested graph neural network and pretrained molecule models. Bioinformatics 40, btae135. 10.1093/bioinformatics/btae135 38449285 PMC10957515

[B21] Trott O. Olson A. J. (2010). Autodock vina: improving the speed and accuracy of docking with a new scoring function, efficient optimization, and multithreading. J. Comput. Chem. 31, 455–461. 10.1002/jcc.21334 19499576 PMC3041641

[B22] van der Maaten L. Hinton G. (2008). Visualizing data using t-sne. J. Mach. Learn. Res. 9, 2579–2605.

[B23] Wang S. Song X. Zhang Y. Zhang K. Liu Y. Ren C. (2023). Msgnn-dta: multi-scale topological feature fusion based on graph neural networks for drug–target binding affinity prediction. Int. J. Mol. Sci. 24, 8326. 10.3390/ijms24098326 37176031 PMC10179712

[B24] Waring M. J. Arrowsmith J. Leach A. R. Leeson P. D. Mandrell S. Owen R. M. (2015). An analysis of the attrition of drug candidates from four major pharmaceutical companies. Nat. Rev. Drug Discov. 14, 475–486. 10.1038/nrd4609 26091267

[B25] Wen M. Zhang Z. Niu S. Sha H. Yang R. Yun Y. (2017). Deep-learning-based drug–target interaction prediction. J. Proteome Res. 16, 1401–1409. 10.1021/acs.jproteome.6b00618 28264154

[B26] Wieder O. Kohlbacher S. Kuenemann M. Garon A. Ducrot P. Seidel T. (2020). A compact review of molecular property prediction with graph neural networks. Drug Discov. Today Technol. 37, 1–12. 10.1016/j.ddtec.2020.11.009 34895648

[B27] Wu H. Liu J. Jiang T. Zou Q. Qi S. Cui Z. (2024). Attentionmgt-dta: a multi-modal drug-target affinity prediction using graph transformer and attention mechanism. Neural Netw. 169, 623–636. 10.1016/j.neunet.2023.11.018 37976593

[B28] Yang H. Chen Y. Zuo Y. Deng Z. Pan X. Shen H.-B. (2024). Mindg: a drug–target interaction prediction method based on an integrated learning algorithm. Bioinformatics 40, btae147. 10.1093/bioinformatics/btae147 38483285 PMC10997434

[B29] Yang Z. Zhong W. Zhao L. Chen C. Y.-C. (2022). Mgraphdta: deep multiscale graph neural network for explainable drug–target binding affinity prediction. Chem. Sci. 13, 816–833. 10.1039/D1SC05180F 35173947 PMC8768884

[B30] Zhang L. Tan J. Han D. Zhu H. (2017). From machine learning to deep learning: progress in machine intelligence for rational drug discovery. Drug Discov. Today 22, 1680–1685. 10.1016/j.drudis.2017.08.010 28881183

[B31] Zhang P. Wei Z. Che C. Jin B. (2022). Deepmgt-dti: transformer network incorporating multilayer graph information for drug–target interaction prediction. Comput. Biol. Med. 142, 105214. 10.1016/j.compbiomed.2022.105214 35030496

[B32] Zhao Q. Zhao H. Zheng K. Wang J. (2022). Hyperattentiondti: improving drug–protein interaction prediction by sequence-based deep learning with attention mechanism. Bioinformatics 38, 655–662. 10.1093/bioinformatics/btab715 34664614

[B33] Zhu H. (2020). Big data and artificial intelligence modeling for drug discovery. Annu. Rev. Pharmacol. Toxicol. 60, 573–589. 10.1146/annurev-pharmtox-010919-023324 31518513 PMC7010403

